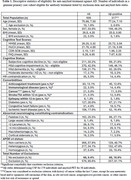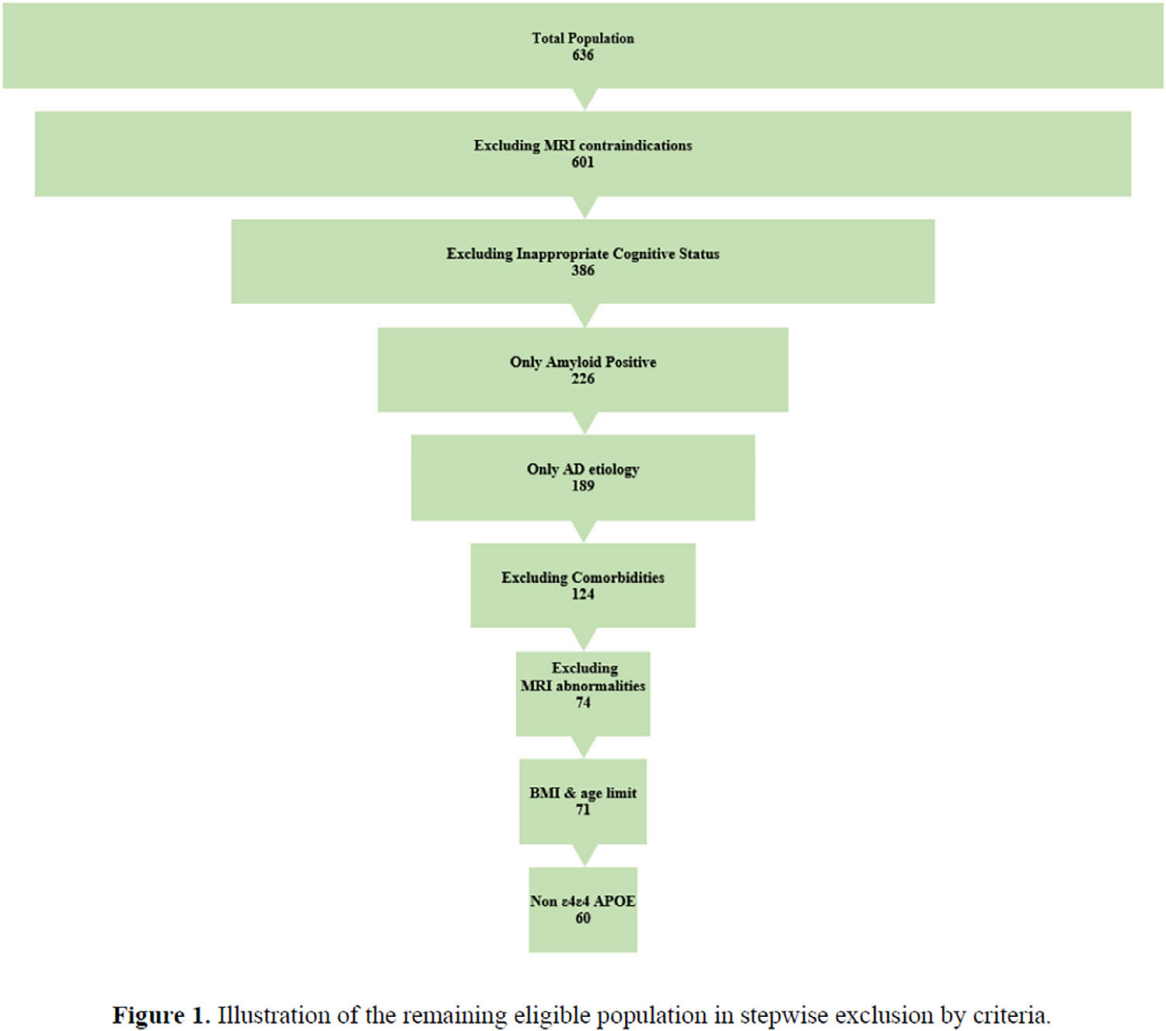# Assessing Eligibility for Anti‐Amyloid Treatment Among Primary Care Patients with Cognitive Symptoms

**DOI:** 10.1002/alz70861_108336

**Published:** 2025-12-23

**Authors:** Josef Erik Barbosa Djärf, Beata Borgström Bolmsjö, Danielle van Westen, Pontus Tideman, Ayesha Fawad, Ruben Smith, Suzanne E. Schindler, Niklas Mattsson‐Carlgren, Erik Stomrud, Oskar Hansson, Sebastian Palmqvist

**Affiliations:** ^1^ Clinical Memory Research Unit, Lund University, Malmö, Skåne Sweden; ^2^ Memory Clinic, Skåne University Hospital, Malmö, Skåne Sweden; ^3^ University Clinic Primary Care Skåne, Lund University, Malmö, Skåne Sweden; ^4^ Center for Primary Health Care Research, Lund University, Malmö, Skåne Sweden; ^5^ Diagnostic Radiology, Department of Clinical Sciences Lund, Lund University, Lund Sweden; ^6^ Imaging and Function, Skåne University Hospital, Lund Sweden; ^7^ Department of Neurology, Washington University School of Medicine, St. Louis, MO USA

## Abstract

**Background:**

Primary care is key to early identification and referral of patients with Alzheimer’s disease (AD). Assessing eligibility for new anti‐amyloid treatment may guide appropriate referrals and prevent overburdening specialized care. In this study we determined eligibility for anti‐amyloid treatment in patients undergoing cognitive evaluation in primary care.

**Method:**

Patients were selected from the BioFINDER‐Primary Care study (NCT06120361), which consecutively enrolls patients undergoing cognitive evaluation at 25 primary care units in Sweden. Between January 2020 and April 2025, patients were comprehensively assessed for co‐morbidities, treatments, clinical symptoms, and cognitive status. Eligibility for anti‐amyloid treatment was evaluated based on Cummings et.al (2023). MRI was assessed by a senior neuroradiologist. Amyloid status was determined using cerebrospinal fluid (CSF) Aβ42/40 ratio or amyloid PET. All patients received a full clinical work‐up at a specialized memory clinic.

**Result:**

A total of 636 participants were examined. The prevalence of treatment contraindications is presented in Table 1. 35 individuals had contraindications for MRI and were excluded. From the remainder, 386 (60.7%) participants had treatment‐appropriate cognitive status (mild cognitive impairment or mild dementia) and among these, 226 (35.5%) were amyloid positive. Of these, 37 were considered to have a non‐AD etiology for their symptoms, leaving 189 for further evaluation. After excluding patients with significant comorbidities (active anticoagulant treatment, immunological disease, cancer, stroke/TIA within 12 months, or epilepsy) 124 (19.5%) remained. Excluding those with MRI abnormalities reduced the sample to 74 (11.6%). Additional exclusion criteria (BMI and age restriction) reduced the eligible participant group to 71 (11.2%). Finally, applying the EMA, but not FDA, restriction excluding APOE ε4 homozygotes (11 remaining individuals) resulted in an eligible population of 60 individuals (9.4%) (Figure 1).

**Conclusion:**

About 9‐11% of patients undergoing cognitive evaluation in primary care are potentially eligible for anti‐amyloid treatment under current clinical guidelines. Major exclusion factors included neuroimaging findings, anticoagulant use, and comorbidities. These findings may guide expectations around treatment uptake and support real‐world health‐economic analyses based on patient eligibility. At AAIC, we will present a proposed workflow to streamline identification of treatment candidates from primary to specialist care.